# Impact of Flag Texture on Tick Sampling Efficiency

**Published:** 2018-12-25

**Authors:** Philippe Gil de Mendonça

**Affiliations:** Institute of Comparative Tropical Medicine and Parasitology, Ludwig Maximilian University, Munich, Germany

**Keywords:** *Ixodes ricinus*, Larval ticks, Nymphal ticks, Flagging

## Abstract

**Background:**

There is a strong interest in tick-borne diseases worldwide due to their negative impact on both human and animal health. Epidemiological studies of tick-borne diseases depend on reliable data on tick population dynamics and activity patterns. Such data are essentially based on tick sampling in the field. This study aimed to evaluate the impact of cloth type on the efficiency of field sampling by the flagging technique.

**Methods:**

The impact of cloth type on the efficiency of field sampling by the flagging technique was investigated by comparing tick sampling yields of two different fabrics, the Munich type (MUC) vs. the Oxford type (OX), based on 30 pairs of transect lines. Data analysis included classical statistics and computer modelling.

**Results:**

The MUC flag yielded nearly five times more larval ticks than the OX flag, whereas the differences in yields for nymphs and adult ticks were not statistically significant based on classical statistics.

**Conclusion:**

The flag made of MUC type fabric, thanks to its tight and relatively flat texture, facilitates detection and collection of ticks from its surface. The OX flag, due to its loose texture, is unsuitable for the quantitative sampling of larval *Ixodes ricinus*.

## Introduction

There is an increasing interest in tick-borne diseases worldwide, as it is believed several of these diseases are emerging and spreading (1, 2). Consequently, various research projects were launched to investigate ticks and tick-borne diseases at regional, national, and international levels. The EU-funded EDEN project focused specifically on investigating emerging zoonoses (3). EDEN actually stands for ‘Emerging Diseases in a changing European eNvironment’. Within this context, ticks were routinely sampled and screened for a palette of zoonotic pathogens throughout most of Europe. To ensure comparability of results, standardized protocols were issued to all participants.

**Fig. 1.**
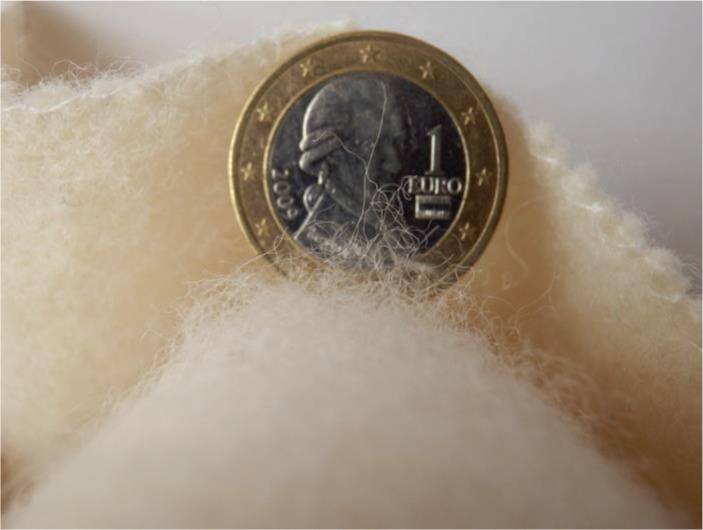
Photograph of the Oxford fabric showing its fleecy texture. The coin used as a size marker is 23.3mm in diameter

A present from Prof SE Randolph (Oxford University) provided the opportunity to compare the standard flannel flag routinely used by the German EDEN team with a totally different type of material, used in other tick studies abroad. Preliminary testing revealed that users experienced difficulties in retrieving ticks embedded in the fibres of this fleecy cloth (Fig. 1). This raised the question of the potential impact of such a different cloth type on tick sampling efficiency. Indeed cloth consistency is known to be a factor influencing flagging efficiency (4), as are flagging distance and pace. This last point introduces an additional factor, the human factor, which includes many components and plays an important part in sampling efficiency.

The test campaign described here therefore aimed to evaluate the impact of cloth type on flagging efficiency by comparing the standard ‘Munich cloth’ with the ‘Oxford cloth’ while limiting the impact of the human factor by removing inter-individual variability.

## Materials and Methods

Questing ticks were sampled in the morning hours by dragging a 1m^2^ flag over soil and vegetation in mixed woodlands including both coniferous and broad-leaved trees with moderate herb and moss layers.

**Fig. 2.**
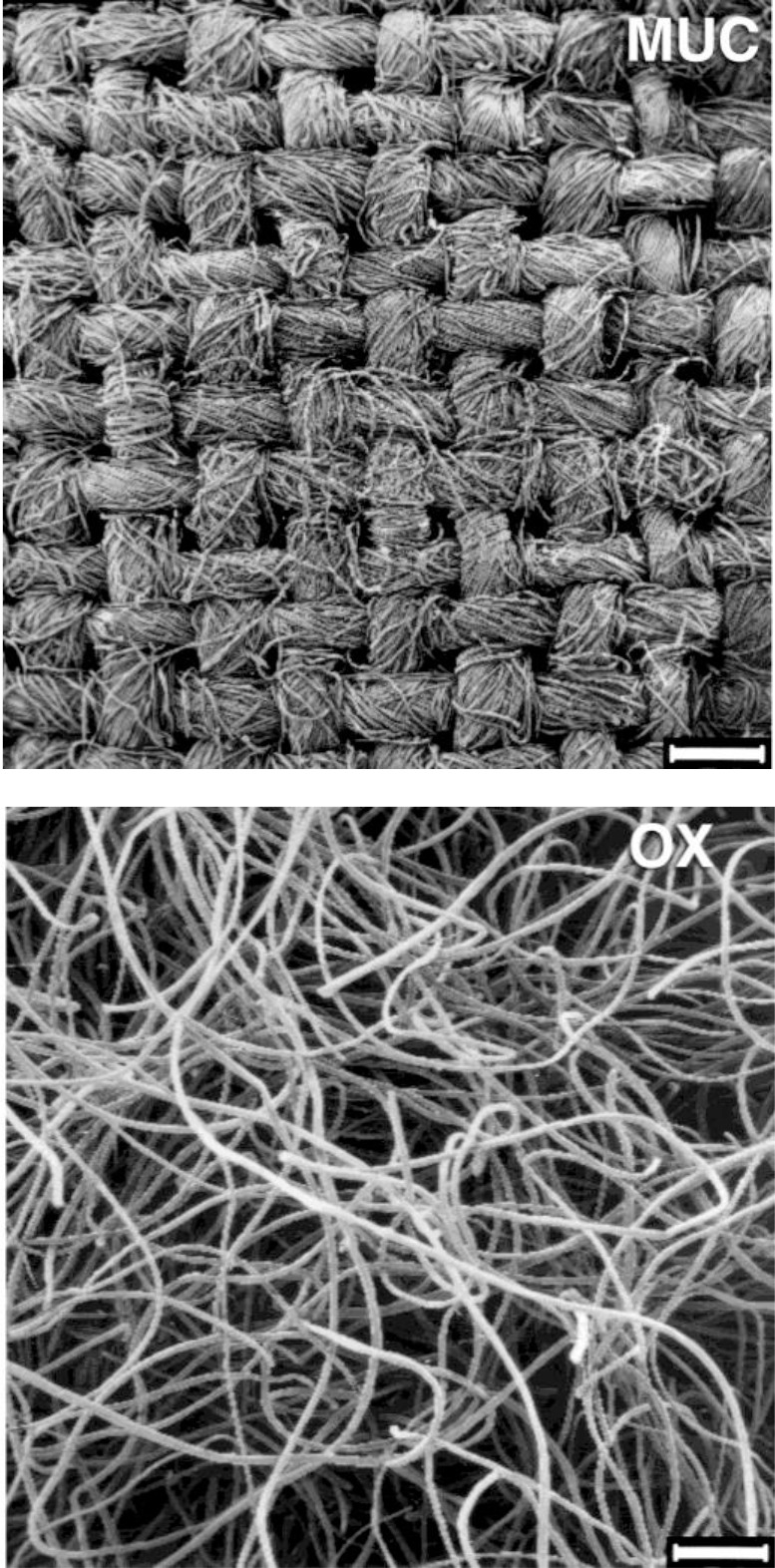
Scanning electron micrographs of the Munich (MUC) and Oxford (OX) fabrics. Scale bars: 500μm

The flag was made of a 1m^2^ removable cloth strung over the terminal section of a long bamboo cane. Two cloth types were available. The Munich type (MUC) was made of a thin and simple white flannel fabric, whereas the Oxford type (OX) was made of a heavier and thicker fabric with a complex structure (Fig. 2).

Two transect lines, close to each other, were surveyed on each sampling session. One transect line was surveyed using the MUC flag, while the other was surveyed using the OX flag. In order to mitigate the effect of timing on sampling efficiency, 15 sampling sessions started with the MUC transect line and ended with the OX transect line, while the other 15 sampling sessions followed the reverse order. Sampling order was based on a computer-generated table of random numbers containing 15 odd numbers and 15 even numbers.

Overall, 30 pairs of transect lines were surveyed by one and the same person at four field sites in the Bavarian districts of Amberg-Sulzbach (site AM1), Landshut (sites LA1 and LA2) and Passau (site PA1) in 2007–2008. Transect lines were divided into 25 segments of 2m^2^ each, thus covering a 50m^2^ surface. At the end of each segment, the flag was inspected and ticks were removed and preserved in 80% ethanol for taxonomic and molecular investigations. Tick identification was based on morphological criteria (5–7).

Before leaving any field sampling site, each cloth was bagged separately in a tight zip locking plastic pouch. Between each of the field sampling trips, each cloth was frozen overnight at −70 °C to kill any tick that might have remained hidden inside the fabric. To avoid condensation on the cloth fibres, no cloth was removed from its plastic pouch until it had regained ambient temperature.

The Mann-Whitney U test, which is suitable for skewed distributions, was applied to statistical comparisons between transect lines. Additionally, computer modelling was performed using raw data from each transect segment rather than cumulative values from transect lines. A computer program was written to perform 10000 Monte Carlo simulations based on a null model.

## Results

Overall, 3957 ticks were collected during this test campaign. Of these, 2587 (i.e. 65.38%) were collected with the MUC flag, while 1370 (i.e. 34.62%) were collected with the OX flag. All ticks were *Ixodes ricinus*. Tick distributions were skewed (s^2^ >m) due to the occurrence of ‘tick nests’, i.e. local concentrations of larvae near their hatching site. The MUC flag yielded 1704 out of 2059 larvae (i.e. 82.76%) while only 355 larvae (i.e. 17.24%) were collected from the OX flag. Between 1 and 455 larvae were collected per transect line using the MUC flag, while transect lines surveyed with the OX flag yielded between 0 and 112 larvae. This difference in yield is very highly significant (P< 0.001). Yields for each of the transect lines, ranked by tick stage, are provided in Table 1. A total of 829 nymphs (out of 1782, i.e. 46.52%) were collected with the MUC flag, while the OX flag yielded 953 nymphs (i.e. 53.48%). Yields per transect line ranged between 2 and 70 nymphs for the MUC flag, and between 7 and 109 nymphs for the OX flag. The differences in yields for nymphs are not statistically significant (P> 0.05) according to the Mann-Whitney U test. However, computer modelling revealed that nymphal yield from the MUC flag is lower than expected under a null model based on similar sampling conditions from similar virtual tick populations (P< 0.05).

**Table 1.** Tick yields for each pair of transect lines, ranked by tick stage and sex

**Larvae (MUC)**	**Larvae (OX)**	**Nymphs (MUC)**	**Nymphs (OX)**	**Females (MUC)**	**Females (OX)**	**Males (MUC)**	**Males (OX)**
1	0	2	8	0	0	0	0
1	1	5	8	0	0	0	0
4	0	6	10	0	0	0	0
5	0	7	13	0	0	0	0
5	1	8	7	0	0	0	0
7	0	9	12	0	0	0	0
7	2	10	24	0	0	0	0
8	2	15	12	0	0	0	0
10	1	16	24	0	0	0	1
11	2	17	24	0	1	0	1
13	0	20	24	0	1	0	1
18	5	21	37	0	1	0	1
19	0	24	13	0	1	0	1
21	22	24	29	0	1	0	1
26	9	25	18	0	2	1	0
26	20	26	16	0	2	1	0
26	41	27	12	0	4	1	0
27	0	27	18	1	0	1	0
27	3	28	35	1	0	1	1
28	7	31	39	1	1	1	2
30	0	35	33	1	2	1	2
33	1	35	47	1	2	1	4
47	1	36	70	1	6	2	0
63	0	39	30	2	1	2	0
74	5	42	41	2	1	2	1
136	2	45	56	2	1	2	2
178	4	58	58	2	2	3	1
185	112	58	109	2	5	3	2
213	95	63	45	4	0	3	3
455	19	70	81	5	2	4	2

Twenty-five adult females and 29 adult males were recovered from the MUC flag, versus 36 adult females and 26 adult males for the OX flag. The differences in yields for adult ticks are not statistically significant (P> 0.05).

## Discussion

The MUC flag yielded nearly five times more larvae than the OX flag. The causes (or at least parts of the causes) for such a dramatic difference were actually observed. The OX cloth is relatively thick and has a very loose and complex texture. Larval ticks, which are small enough, manage to crawl between the fibres of the OX flag. Therefore, although larvae were actually sampled by the flag, many of them were not found (and thus not collected) by the observer because these larvae had become invisible and unreachable deep inside the fabric. On several occasions, larvae were observed crawling out of the cloth on the reverse side, indicating that larvae were able to cross through the cloth to eventually escape. These observations raise a health and safety issue. Indeed, ticks hidden inside the cloth may inadvertently be transported from their original sampling site to other locations, thus possibly transferring tick-borne pathogens to new areas or closer to humans.

**Fig. 3.**
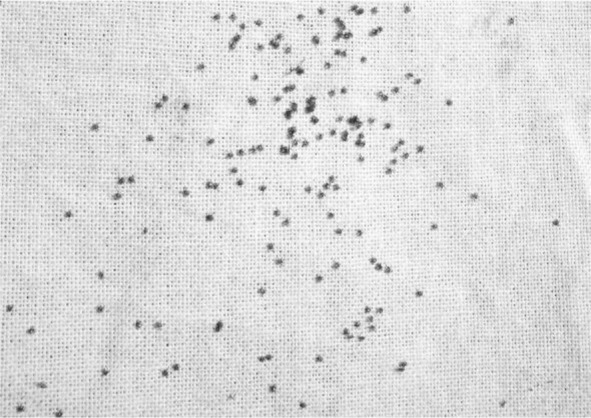
Numerous larval *Ixodes ricinus* ticks are clearly visible to the naked eye on this photograph of a portion of a MUC type flag

In stark contrast to the OX fabric, the MUC fabric presents a relatively smooth and flat surface. The fibres are tight enough to prevent larval ticks from crawling through them. Larvae have nowhere to hide and cannot escape through the cloth. Thanks to the white and flat surface, larvae are easily detected visually (Fig. 3) and easily collected.

A trend suggesting there might be a difference in efficiency at sampling nymphs between the MUC and OX flags was observed. Based on classical statistics, this trend was not statistically significant. However, computer modelling suggests that the MUC flag might be marginally less efficient at sampling nymphs than the OX flag, which is consistent with the above-mentioned trend.

The aforementioned differences in sampling efficiency for larvae and (to a much lesser extent) nymphs clearly have an impact on estimates of questing activity for these two developmental stages. Studies relying heavily on such estimates, e.g. studies of cofeeding potential (and its implications for disease transmission) between larvae and nymphs, will undoubtedly suffer biases due to heavily underestimated larval activity when using the OX cloth.

The validity of comparisons between various studies needs to be questioned when no indications of relative sampling efficiency are provided, particularly when larvae are considered.

The nearly five times difference in yield observed here for larval ticks using two different cloth types is an extreme example. However, this extreme example serves well to illustrate an important point: In large research projects spread over wide geographic areas where equipment is purchased from local providers, it is wise to perform some preliminary comparative testing as part of internal quality control prior to engaging in large-scale sampling. Indeed, locally purchased material may vary to a non-negligible extent from place to place, thus potentially introducing biases in the study. Computer modelling based on preliminary test results should help highlight potential biases. Indeed, Monte Carlo methods are known to have as much power as (and often more power than) classical statistical tests, while offering a wider flexibility of use (8).

## Conclusion

The flag made of MUC type fabric, thanks to its tight and relatively flat texture, facilitates detection and collection of ticks from its surface. The OX flag, due to its loose texture, is unsuitable for the quantitative sampling of larval *I. ricinus*, as these can crawl between the fibres to hide inside the fabric itself and eventually escape. Consequently, the OX flag also presents a potential health and safety issue due to the risk of transporting ticks and transferring tick-borne pathogens to new areas or closer to humans.
